# Alpha, Beta and Gamma Diversity Differ in Response to Precipitation in the Inner Mongolia Grassland

**DOI:** 10.1371/journal.pone.0093518

**Published:** 2014-03-27

**Authors:** Qing Zhang, Xiangyang Hou, Frank Yonghong Li, Jianming Niu, Yanlin Zhou, Yong Ding, Liqing Zhao, Xin Li, Wenjing Ma, Sarula Kang

**Affiliations:** 1 School of Life Sciences, Inner Mongolia University, Hohhot, China; 2 Grassland Research Institute of Chinese Academic of Agricultural Science, Hohhot, China; 3 Sino-US Center for Conservation, Energy and Sustainability Science, Inner Mongolia University, Hohhot, China; 4 AgResearch Grasslands Research Centre, Palmerston North, New Zealand; Tennessee State University, United States of America

## Abstract

Understanding the distribution pattern and maintenance mechanism of species diversity along environmental gradients is essential for developing biodiversity conservation strategies under environmental change. We have surveyed the species diversity at 192 vegetation sites across different steppe zones in Inner Mongolia, China. We analysed the total species diversity (γ diversity) and its composition (α diversity and β diversity) of different steppe types, and their changes along a precipitation gradient. Our results showed that (i) β diversity contributed more than α diversity to the total (γ) diversity in the Inner Mongolia grassland; the contribution of β diversity increased with precipitation, thus the species-rich (meadow steppe) grassland had greater contribution of β diversity than species-poor (desert steppe) grassland. (ii) All α, β and γ species diversity increased significantly (*P*<0.05) with precipitation, but their sensitivity to precipitation (diversity change per mm precipitation increase) was different between the steppe types. The sensitivity of α diversity of different steppe community types was negatively (*P*<0.05) correlated with mean annual precipitation, whereas the sensitivity of β and γ diversity showed no trend along the precipitation gradient (*P*>0.10). (iii) The α diversity increased logarithmically, while β diversity increased exponentially, with γ diversity. Our results suggest that for local species diversity patterns, the site species pool is more important in lower precipitation areas, while local ecological processes are more important in high precipitation areas. In addition, for β diversity maintenance niche processes and diffusion processes are more important in low and high precipitation areas, respectively. Our results imply that a policy of “multiple small reserves” is better than one of a “single large reserve” for conserving species diversity of a steppe ecosystem, and indicate an urgent need to develop management strategies for climate-sensitive desert steppe ecosystem.

## Introduction

Half a century ago, Whittaker first defined species diversity from three different perspectives: alpha (α) diversity, beta (β) diversity, and gamma (γ) diversity [1]. Alpha and γ diversity are grouped as inventory diversity [2], sharing the same characteristics and differentiated only by scale. Beta diversity is defined as the difference in species composition between communities and is closely related to many facets of ecology and evolutionary biology [3], [4], [5]. Most research on species diversity has focused on inventory diversity, but research on β diversity has recently increased [3], [6], [7]. The distribution patterns and maintenance mechanisms of species diversity along environmental gradients have been core topics in ecological research [4], [8], [9]. It is essential to understand such patterns and mechanisms for the development of strategies and measures for conserving species diversity under environmental change. Whether or not a single large or several small (SLOSS) reserves are superior means of conserving biodiversity [10] depends on the dominant type of diversity present. For example, a high β species diversity within a community type may theoretically imply that the community occupies a heterogeneous environment. In that situation, the use of the ‘several small’ strategy will be superior to the “single large” strategy in reserve design for species diversity conservation [11].

Studies on the geographical patterns of species diversity generally showed a decreasing trend of α and γ diversity with increasing latitude from tropics to poles, which was primarily driven by temperature [8], [12]. However, the correlations between β diversity and latitude were inconsistent; they could be positively [13], [14], negatively [15], [16], or not correlated [9], [17]. Numerous studies have also reported species diversity patterns along other environmental gradients [18], [19], [20], and in many cases the species diversity-precipitation relationships have been studied [21], [22], [23]. Precipitation is the most important factor affecting species diversity and ecosystem functioning in arid and semiarid grasslands, such as in the Eurasian steppe, North American prairie and African savanna [23], [24], [25]. However, reported relationships between species diversity and precipitation have been inconsistent for grassland biomes and scales; in some cases increasing precipitation promoted species diversity [21], [22], [26], but in other cases it had little effect [21], [25].

Species evolution and diffusion, inter-specific competition, and environmental changes commonly influence the α, β and γ diversity of plant communities, but the response of species diversity pattern to these biological and environmental changes and the mechanisms for the response differ among the three species diversity measures [27], [28], [29], [30]. Climate, geological history and ecological randomness are considered to be the main factors affecting species diversity pattern at large scales [31], [32], whereas the local ecological processes and regional species pool are considered to be important at a small scale [33], [34]. Environmental heterogeneity and species diffusion are closely related to β diversity [9], [35]. The relative importance of different components of species diversity in maintaining the total species diversity differs with the spatial and temporal scales or across different regions [15], [36].

The Inner Mongolia steppe grassland is an essential part of the Eurasian steppe [37]. Owing to the heterogeneous environment and the particular geological and evolutionary history, the grassland is rich in plant species diversity. It has more than 2,300 vascular plant species [38], and is recognised as the second most species-rich grassland biome in the world in terms of indigenous plant biodiversity after the African savanna [39]. The Inner Mongolia grassland is one of the key areas for biodiversity conservation in the world. The grassland types in Inner Mongolia show clear zones along a climatic gradient, from the desert steppe in dry areas, to the typical steppe, and through to the meadow steppe [40], [41]. There have been several studies on the effects of precipitation on the species diversity and grassland productivity in the Inner Mongolia [24], [42], but the composition of species diversity (α, β, γ) and their changes across different steppe grassland types on environmental gradients has not been studied. In the present study, we measured the components of species diversity (α, β, γ) in each of the major steppe community types. The aim of the study was to present the composition of, and the relationships between α, β, and γ diversity of main steppes types in Inner Mongolia, and analyse the pattern of variation along the precipitation gradient. Then, based on these diversity patterns and relationships, we discuss the mechanism of species diversity maintenance in the Inner Mongolia grassland, and suggest the best strategies for species diversity conservation under environmental change.

## Methods

### Ethics statement

All the survey sites were owned and/or managed by pastoral farmers, who gave permission to the survey. The field studies did not involve any endangered or protected species.

### Study area

**Figure 1:**
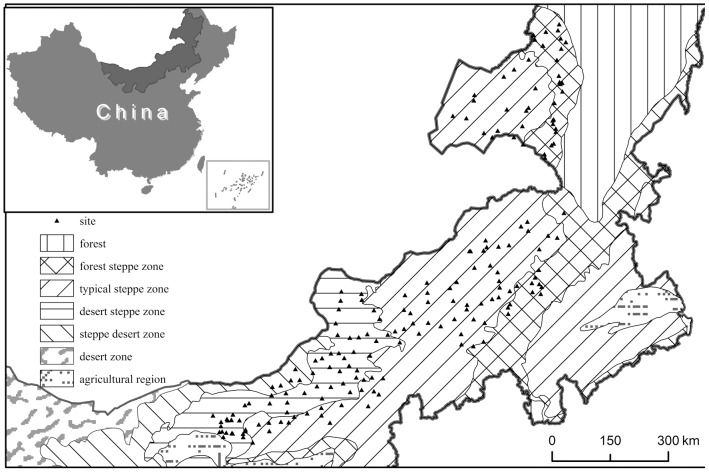
Map of the study region, showing the vegetation zones and sampling sites.

We surveyed the grassland species diversity in the whole region of the Inner Mongolia grassland in northern China. The region stretches from latitudes 41.31°N to 50.78°N and longitudes 108.16°E to 120.39°E with elevation ranging from 532 to 1725 m above sea level (Fig. 1). The typical landforms in this region include gently rolling plains, tablelands, and hills. Mean annual temperature (MAT) ranges from −3.0 to 6.7°C, and mean annual precipitation (MAP) varies from about 150 to 450 mm, decreasing from southeast to northwest [43]. Distributed along the climate gradient from the relatively humid southeast to relatively dry northewest are soil types that very from chernozems, to chestnut- and then calcic brown soils; and grassland biomes that vary from meadow steppe, to typical steppe and then desert steppe grassland (Fig. 1).

### Data collection

We investigated species diversity on 192 sites in the grassland region from late July to mid-August in 2012, when the grassland community biomass was at its peak. Seven dominant steppe community types were investigated: *Leymus chinensis* meadow steppe, *Stipa baicalensis* meadow steppe, *S. grandis* typical steppe, *L. chinensis* typical steppe, *S. krylovii* typical steppe, *S. breviflora* desert steppe, and *S. klemenzii* desert steppe. Nineteen to 39 sites were surveyed for each community type; the number of sites for each type was approximately determined by the relative size of its distribution area. The position of each site was located using GPS (Fig. 1). To focus on the relationships between species diversity and climate, and to minimize the influence of domestic animal grazing, all the surveyed sites were selected either in fenced grassland under protection, or in mowed grassland (surveyed before haymaking at the end of August). Grassland sites with obvious grazing effects (recognised by species composition) were excluded from the survey. At each site an area of 10×10 m was delineated, and ten 1×1 m plots were randomly placed in the delineated area to record all the plant species.

Meteorological records from the 156 meteorological stations in the study area were used in the analysis of the diversity-precipitation relations [44].

### Data analyses

#### Calculation of species diversity

Since the concept of β diversity was introduced in 1972 [45], more than 40 different methods have been proposed for its calculation [6], [46]. Jurasinski [2] suggested that these calculation methods could be split into two groups: the first group, designated differentiation diversity, includes a similarity coefficient, similarity attenuation slope with distance, gradient length in ordination space, and total variance of community composition [9], [47]. The second group, designated proportional diversity, includes additive partition diversity and multiplicative partitioning diversity [28], [45]. Additive diversity partition expresses α and β diversity in the same unit so that their relative importance can be easily quantified and interpreted, and can be directly compared across spatial and temporal scales or land-use practices [28], [34], [48]. We used the additive partition approach to calculate total species diversity of the studied grassland and its components. The methods were described in [27], [28], and are briefly described below:

- *α* diversity, also called community diversity, is defined as the mean of the species richness (number) in the surveyed ten plots at a site:
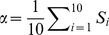
(Eq.1)where *S_i_* represents the species richness in each plot.
- γ diversity, also called site diversity, is the total species richness at a site.
- *β* diversity, defined as the difference in species composition among the ten plots at a site, is calculated by subtracting α diversity from *γ* diversity:
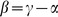
(Eq.2)The *β* diversity has two components, one quantifying the degree of nestedness of species richness (*β_N_*), *i.e*. the degree to which species richness differs between plots within one site from the most species-rich plot, and the other component reflecting the difference in species composition among the plots (*β_R_*). With *S_max_* representing the species number in the richest plot, *β_N_* and *β_R_* are calculated as follows:
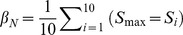
(Eq.3)

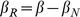
(Eq.4)

Consequently, the total species richness at a site (*γ*) is the sum of the mean species richness of all the plots at the site (*α*), the differences in species richness due to the nestedness (*β_N_*) and species composition (*β_R_*) among the plots:
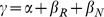
(Eq.5)


The components of species diversity (*α*, *β* and *γ*) were assessed for each grassland site. The species diversity at all of the sites for each steppe community type was averaged to represent the species diversity of the community type. The species diversity composition of three steppe vegetation types (i.e., the desert steppe, typical steppe and meadow steppe) were also aggregated in the same way to represent the species diversity at all grassland sites of a steppe vegetation type.

#### Calculation of precipitation at each site and for each grassland type

We calculated mean annual precipitation (MAP) at each vegetation site using the approach of Thornthwaite [49] based on precipitation records of the 156 meteorological stations in the region. Each site MAP was derived according to the latitude (LAT), longitude (LNG) and altitude (ALT) of the site, using a previously developed model on the relationship between MAP and the geographical coordinates of each meteorological station [44]:
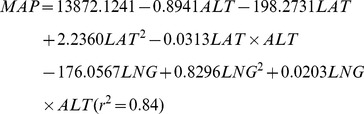
(Eq.6)


The MAP of each community type was calculated as the mean of the MAP at all the sites that belongs to the community type.

#### Relationships among species diversity components

First, the proportions of α and β to γ diversity were used to quantify the relative contribution of these two kinds of diversity to γ diversity. The composition of γ diversity (i.e., the proportions of α, β, β_R_ and β_N_) of the seven steppe community types, and of the three steppe vegetation types, was ordered according to the precipitation of their distribution areas in order to examine the species diversity composition changes across these steppe types in relation to precipitation.

Second, we used regression analysis to model relationships between γ diversity and its components (α and *β*) across all 192 sites. The relationships between γ diversity and the ‘occasional species’ (species recorded in only one or two of the ten plots at a site) were also analysed. These relationships were used to interpret the mechanism of species diversity maintenance in the grasslands.

#### Patterns of species diversity along a precipitation gradient

With α, β and γ diversity and the precipitation (MAP) calculated for all the vegetation sites, we plotted each diversity measure against precipitation, and tested for a significant correlation between species diversity and MAP using linear regression analysis. We also did a linear regression analysis between the species diversity and precipitation for each of the seven steppe community types, and used the slope of the regression (i.e. diversity change per mm precipitation change) to represent the sensitivity of the species diversity of each community type to precipitation. A steep slope or high sensitivity means a greater effect of precipitation on community species diversity. The changes in sensitivity across seven community types along a precipitation gradient were tested by examining if the sensitivity and precipitation of these community types were significantly correlated.

All statistical analyses were performed using Excel 2010 and SPSS 17.0.

## Results

### Species diversity and diversity composition of the Inner Mongolia grassland

**Figure 2:**
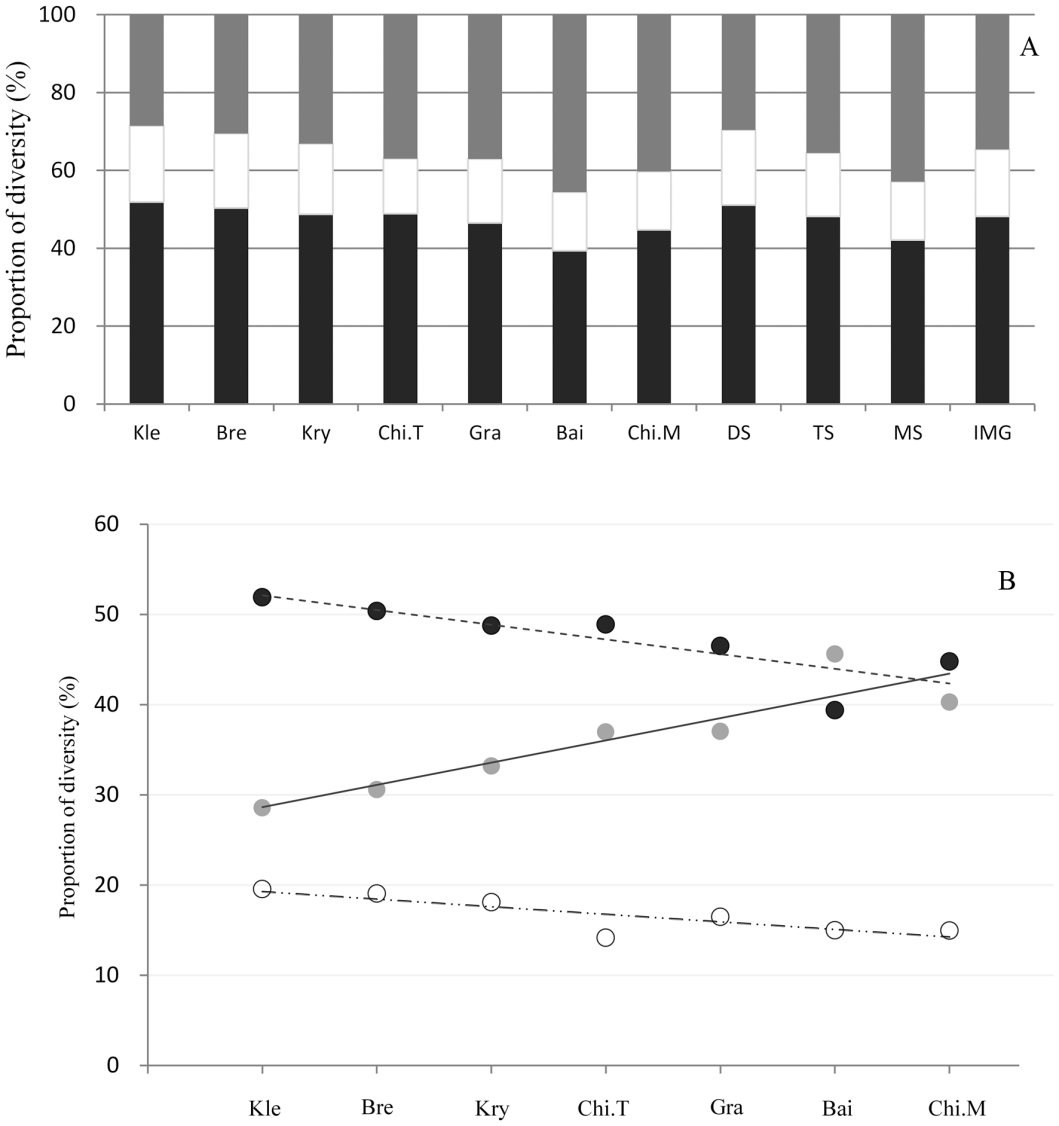
Species diversity of the steppe grassland in Inner Mongolia. The data are presented for seven major steppe community types: Kle (*Stipa klemenzii* desert steppe), Bre (*Stipa breviflora* desert steppe), Kry (*Stipa krylovii* typical steppe), Chi.T (*Leymus chinensis* typical steppe), Gra (*Stipa grandis* typical steppe), Bai (*Stipa baicalensis* meadow steppe), Chi.M (*Leymus chinensis* meadow steppe); three steppe vegetation types: DS (desert steppe), TS (typical steppe), MS (meadow steppe); and the Inner Mongolia grassland as a whole (IMG). A: Proportion of *α* diversity (black), *β* nestedness diversity (*β_N_*) (white) and *β* replacement diversity (*β_R_*) (grey) in *γ* diversity; B: The trend of the proportion of *α* diversity (black dot), *β_N_* diversity (white dot) and *β_R_* diversity (grey dot) in *γ* diversity in the seven grassland types with the types ordered according to the annual mean precipitation of their distribution areas, with precipitation increase from left to right.

The species diversity (*γ*) and its components (*α*, *β_R_* and *β_N_*) were consistently low in desert steppe and high in meadow steppe, and in-between in the typical steppe (Table 1). The *β* diversity had a slightly greater contribution (51.83%) to *γ* diversity than α diversity (48.17%) in the Inner Mongolia grassland (Table 1 and Fig. 2A). The contribution of *α* and *β* diversity to *γ* diversity differs among the seven steppe community types and among the three steppe vegetation types (Fig. 2A); the contribution of *α* diversity was higher in desert steppe than in meadow steppe, and conversely the contribution of *β* diversity was lower in desert steppe than in meadow steppe. The change in the contribution of *β* diversity among the steppe community types was mainly due to changes in *β* replacement diversity (*β_R_*), while *β* nestedness diversity (*β_N_*) showed little change. When these steppe types were ordered according to the MAP of their distribution areas, a trend of decreasing *α* and increasing *β* with increasing MAP was shown (Fig. 2B).

**Table 1:** The environmental characteristics (mean annual precipitation MAP, mean annual temperature MAT, and major soil types) and species diversity composition (α, β and γ) of the seven steppe community types and of the three vegetation types in the Inner Mongolia grassland.

Type	No. of sites	MAP	MAT	Major soil types	*α*	*β*	*γ*	*β_N_*	*β_R_*	*α%*	*β%*
*Stipa klemenzii* desert steppe	36	196	3.52	calcic brown soil	9.5±0.3	9.0±0.4	18±0.7	3.6±0.2	5.4±0.4	51.9±0.9	48.1±0.9
*Stipa breviflora* desert steppe	39	212	3.18	Calcic brown and light chestnut soil	10.3±0.3	10.4±0.5	20.7±0.6	3.9±0.2	6.5±0.4	50.4±1.2	49.6±1.2
*Stipa krylovii* typical steppe	31	263	0.85	chestnut and light chestnut soil	12.8±0.4	13.7±0.6	26.4±0.9	4.7±0.2	8.9±0.5	48.7±0.6	51.3±0.6
*Leymus chinensis* typical steppe	28	305	0.15	chestnut and dark chestnut soil	12.6±0.4	13.5±0.7	26.1±1.0	3.7±0.2	9.8±0.6	48.9±0.9	51.1±0.9
*Stipa grandis* typical steppe	21	329	−0.40	chestnut soil	15.1±0.5	17.8±0.8	32.8±1.3	5.3±0.2	12.4±0.7	46.5±0.8	53.5±0.8
*Stipa baicalensis* meadow steppe	18	348	−2.02	chernozem and light chernozem soil	20.7±0.8	32.3±1.7	53.0±2.5	7.8±0.4	24.5±1.5	39.4±0.9	60.6±0.9
*Leymus chinensis* meadow steppe	19	354	−1.85	chernozem soil	18.2±0.7	22.6±0.9	40.8±1.3	6.0±0.4	16.5±0.8	44.8±1.1	55.2±1.1
Desert steppe	75	205	3.34	mainly on calcic brown soil	9.9±0.2	9.7±0.3	19.6±0.5	3.7±0.2	5.97±0.3	51.1±0.7	48.9±0.7
Typical steppe	80	302	0.27	mainly on chestnut soil	13.3±0.3	14.7±0.5	28.0±0.7	4.5±0.2	10.2±0.4	48.2±0.5	51.8±0.5
Meadow steppe	37	351	−1.93	mainly on chernozem soil	19.4±0.5	27.3±0.2	46.7±1.5	6.9±0.4	20.4±1.0	42.2±0.8	57.9±0.8
Inner Mongolia grassland	192	273	1.05	all the soils above	13.1±0.3	15.2±0.6	28.3±0.8	4.7±0.1	10.5±0.5	48.2±0.4	51.8±0.4

The β diversity has two components of β nestedness diversity (β_N_) and β replacement diversity (β_R_). The diversity values are the mean ± s.e.m of species number recorded in ten plots at each site. The percentage of α and β diversity in γ diversity are also shown as α% and β%.

### Species diversity pattern and their components along a precipitation gradient

**Figure 3:**
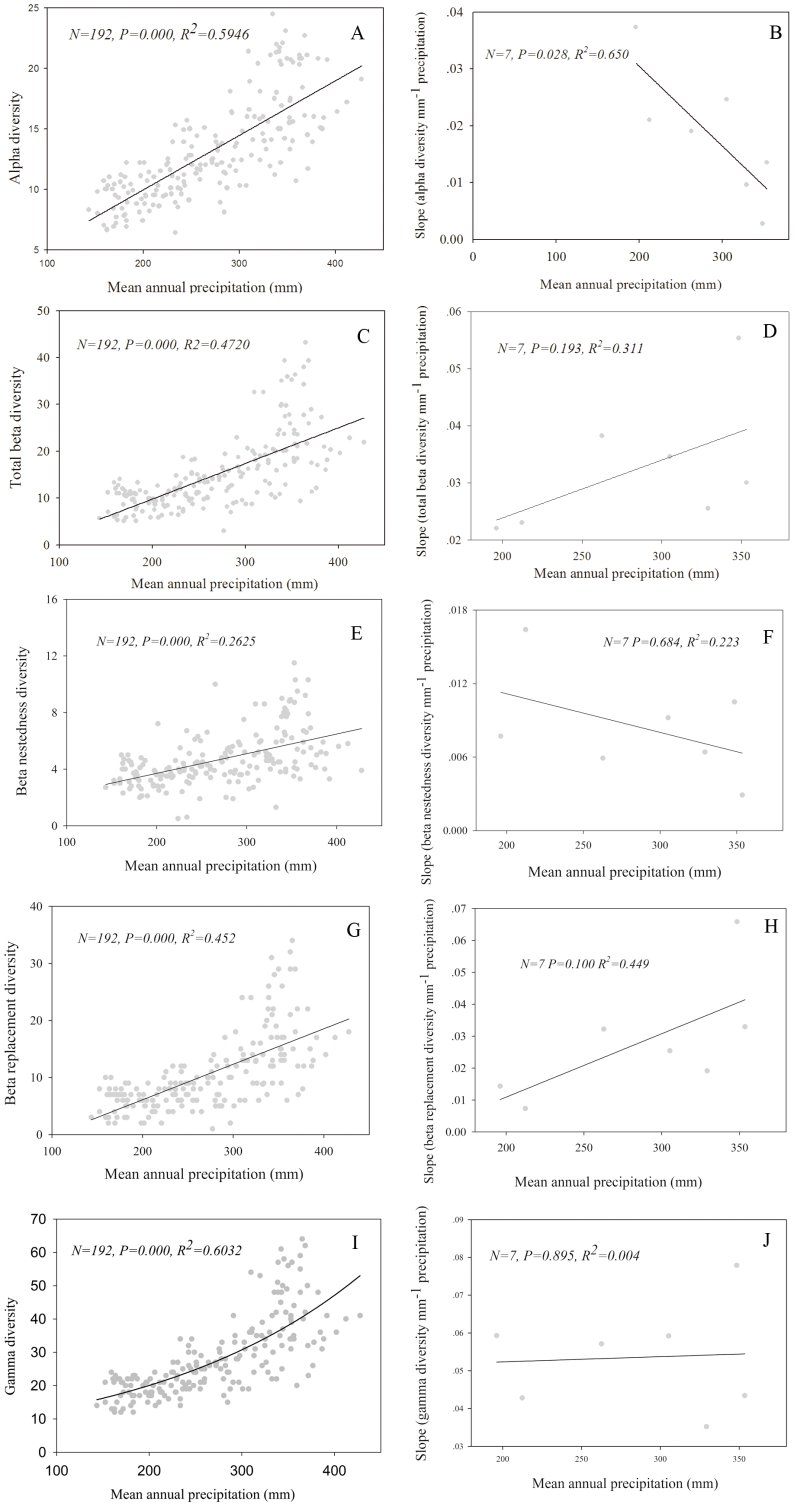
Response of species diversity to precipitation (left column), and the sensitivity of species diversity of seven steppe community types to precipitation change (right column) in the Inner Mongolia grassland. The sensitivity is represented by the regression slope of the linear regression of species diversity to precipitation within each steppe type. A: response of *α* diversity; B: sensitivity of *α* diversity; C: response of *β* diversity; D: sensitivity of *β* diversity; E: response of *β_N_* diversity; F: sensitivity of *β_N_* diversity; G: response of *β_R_* diversity; H: sensitivity of *β_R_* diversity; I: response of *γ* diversity; J: sensitivity of *γ* diversity.

The *α* diversity increased significantly (P<0.001) with increasing precipitation (Fig. 3A), but the sensitivity of *α* diversity to precipitation across the seven steppe community types significantly decreased (P = 0.028) with increasing precipitation (Fig. 3B).

Both *β_N_* diversity and *β_R_* diversity increased with precipitation, and consequently total *β* diversity also increased (Fig. 3C, E, G). However, there was no correlation between the sensitivity of *β*, *β_N_* or *β_R_* diversity to precipitation across the seven community types along the precipitation gradient (P>0.05), though the sensitivity of *β_R_* diversity showed a non-significant increasing trend (P = 0.10) (Fig. 3D, F, H).

The *γ* diversity also significantly increased with precipitation (Fig. 3I), but the sensitivity of *γ* diversity to precipitation showed no correlation with MAP across the seven community types (Fig. 3J).

### Relations among the γ diversity and its components

**Figure 4:**
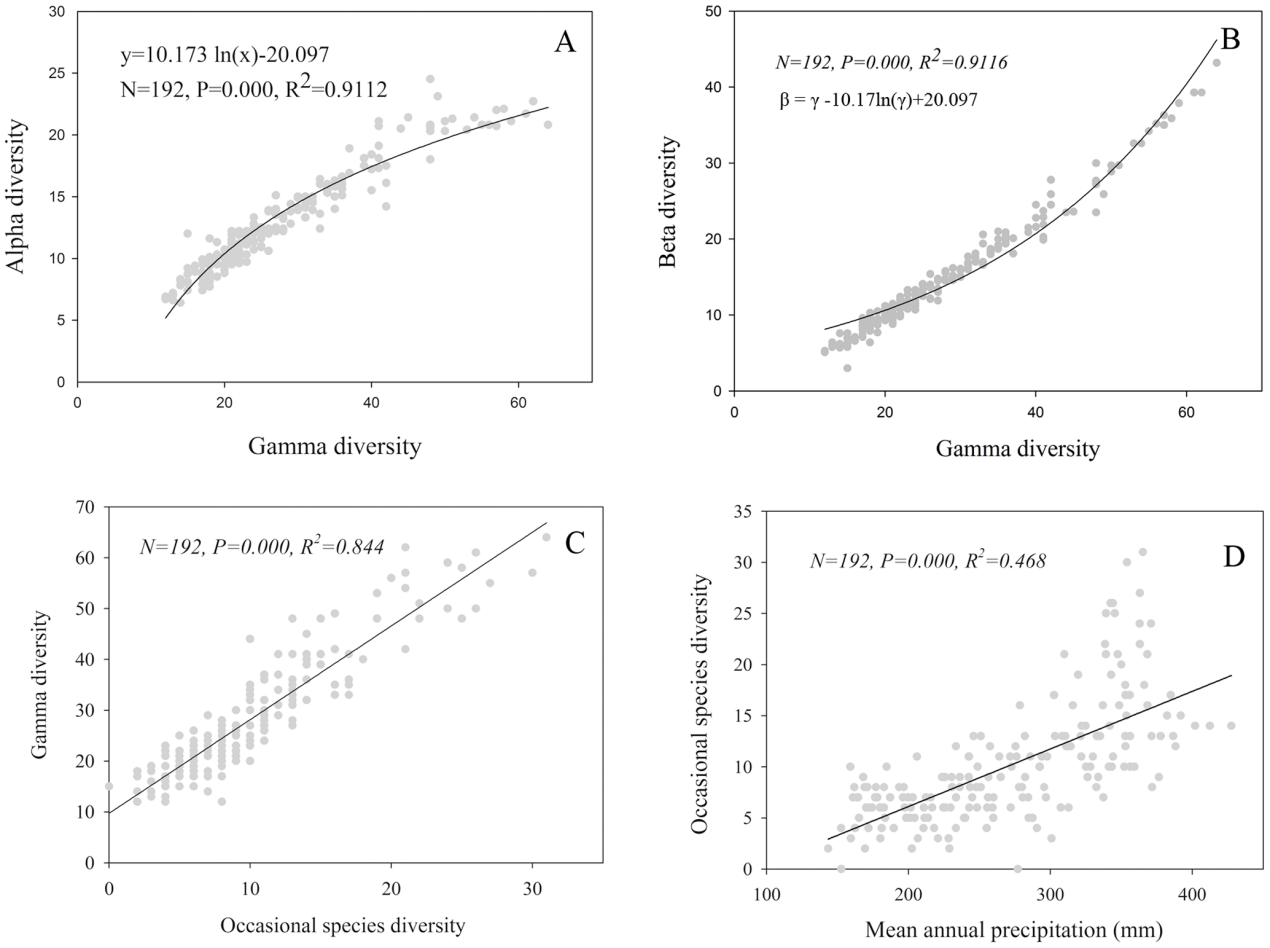
Relations of *α* diversity (A), *β* diversity (B) and occasional diversity (C) with *γ* diversity in the Inner Mongolia grassland. The *β* diversity is complementary to *α* diversity in *γ* diversity, and the curve in (B) is derived as *β*  =  *γ* – α. D: response of occasional species diversity to mean annual precipitation.

The α diversity increased with γ diversity, and the increase was gradually saturates and could be described by a logarithmic curve (P<0.001) (Fig. 4A). The β diversity also increased with γ diversity, but the increase was, complementarily to α diversity, accelerated with the increase of γ diversity (Fig. 4B). There was a significant linear correlation between *γ* diversity and occasional species diversity (Fig. 4C). The occasional species diversity also increased significantly (P<0.001) with increasing precipitation (Fig. 4D).

## Discussion

### Contribution of *α* and *β* to *γ* diversity changes across different steppe types along a precipitation gradient

The contributions of *α* and *β* diversity to *γ* diversity form the basis for understanding the biodiversity components [4], [46]. Controversial opinions exist on the relative importance of *α* and *β* diversity to *γ* diversity: some believe that *α* diversity is more important, while the others contend that β diversity is more important. A third group suggest that *α* and *β* diversity work together [4], [27], [46]. We have found that *β* diversity contributes slightly more (51.83%) than *α* diversity (48.17%) to *γ* diversity in the Inner Mongolia grassland (Fig. 2), and that contribution of *β* diversity is greater in species-rich grassland (meadow steppe) in high precipitation areas than in species-poor grassland (desert steppe) in low precipitation areas. In other words, the contribution of *β* diversity has an increasing trend with precipitation (Fig. 2B).

The relative contribution of *α* and *β* to *γ* diversity in biological communities depends on the ecological heterogeneity and capability of species diffusion [27], [28]. The *α* diversity is more important in communities with an homogeneous environment and strong-diffusion species, whereas *β* diversity is, on the contrary, more important in communities with an heterogeneous environment and weak-diffusion species. The increase of ecological heterogeneity with increasing precipitation in the studied grassland region [22], [42] may be attributable to the increase in the contribution of *β* diversity in *γ* diversity with precipitation increase. This is supported by the high occasional species diversity in the species-rich grasslands (Fig. 4C). High occasional species diversity is also in accordance with high species replacement diversity (*β_R_*) (Fig. 2). Thus, the increase of the contribution of *β* to *γ* diversity along the gradient of increasing precipitation is most likely associated with the increase in ecological heterogeneity and the decrease in species diffusion.

### Maintenance mechanism of species diversity along a precipitation gradient changes from regional species pool to the effect of local ecological processes

The maintenance mechanism of local species diversity has always been an important topic in ecology. Local ecological processes (such as predation, competition, resource supply and diffusion) [50], [51] and the regional species pool [33], [34] have been considered as the mechanisms for diversity maintenance. However, there is insufficient understanding on which of these two mechanisms is more important for local species diversity patterns [36]. Examining the relationship between regional and local species diversity can help quantify the importance of these two mechanisms [34]. A linear correlation between *γ* diversity and *α* diversity would suggest that the regional species pool was the main limiting factor. Alternatively, a saturated curve between α and γ diversity would suggest ecological processes are more important in maintaining diversity [34].

In the studied grassland, both *α* and *γ* diversity increased as precipitation increased (Fig. 3), but the increase of *α* diversity was gradually saturated (a logarithmical increase with *γ* diversity) (Fig. 4A). That is, the increase of *α* diversity with increasing *γ* increasing was rapid and approximately linear when *γ* diversity was low, but the increase slowed down when *γ* diversity was high in high precipitation areas. In species-poor grasslands (low *γ*) with low precipitation, the inter-specific competition is relatively weak resulting in much spare niche capacity. Niche theory indicates that every species occupies its unique corresponding niche [52], thus an increasing regional species pool provides the possibility for species to occupy more niches in the community. The near linear increase of *γ* diversity with *α* diversity (Fig. 4A) in low precipitation areas may indicate the importance of the regional species pool in determining local species diversity. In the high precipitation grassland region, more species appeared in the community, and inter-specific competition was relatively strong. In the case of full use of resources, the ecological niche was occupied to its fullest extent [53]. This means there was very little spare niche capacity, and no new species could be present in the community. Therefore, the regional species pool was still increasing, but local species diversity was saturated. In other words, with an increase in mean annual precipitation, the dominant maintenance mechanism of community diversity changed from the regional species pool to the effect of local ecological processes.

### The maintenance mechanism of *β* diversity changes from niche processes to diffusion processes with increasing precipitation

The *β* diversity reflects different degrees of species composition. The environment heterogeneity (or the niche process [1], [45]) and species diffusion processes [54] have been recognised as the main mechanisms that combine to maintain *β* diversity [9], [29]. The relative importance of the two processes varies across regions and scales [15], [35]. The nestedness species diversity (*β_N_*) represents the degree to which species richness in each plot differs from the richest plot at a site, and reflects the extent of the variation in species number within each community. It is closely related to resource heterogeneity or the niche processes [27]. In low precipitation areas (*i.e*., desert steppe), strong winds may erode and move the soil from grass-dominant areas to areas occupied by shrub clamps to create fertile “islands”, thus increasing the environmental heterogeneity [55]. In high precipitation areas (*i.e.*, meadow steppe), *β_N_* diversity increased with an increasing species pool. However, since *α* diversity is almost saturated with respect to *γ* diversity (Fig. 4A), *β_N_* diversity does not increase at the same rate as *γ* diversity, resulting in a slight reduction in the proportion of *β_N_* in species-rich grasslands in high precipitation areas (Fig. 2B).

On the contrary, the strong increase of *β_R_* with increasing precipitation (Fig. 4G) indicates a strong species-replacement effect in high precipitation areas [27], [28]. Communities with more weak-diffusing species, such as those recorded as occasional species, tend to form high *β_R_* diversity [27], [35]. Considering the increase of occasional species diversity with precipitation (Fig. 4D) and with *γ* diversity (Fig. 4C), *β_R_* is much more important in the diversity of species-rich than species-poor grassland communities. In summary, the dominant mechanism for *β* diversity maintenance changes from niche processes to diffusion processes across steppe community types from species-poor desert steppe to species-rich meadow steppe along precipitation gradient.

### Management implications

Changes in regional precipitation patterns under global climate change will undoubtedly affect species diversity and ecosystem function and stability [56], [57], and the effects will be especially profound in arid and semiarid grassland regions [24], [58]. Our results have important implications for understanding the potential effects of climate change on the semiarid grassland, and for developing biodiversity conservation strategies. First, the generally greater contribution of *β* diversity than *α* diversity to γ diversity (Fig. 2) implies it is better to construct several small reserves than a single large reserve for protecting species diversity of a steppe community in the region. The much greater contribution of *β* diversity in species-rich (meadow steppe) than in species-poor (desert steppe) communities suggests it more useful to apply multiple small reserves for protecting the meadow steppe grassland in relatively humid areas. Second, since species diversity provides a mechanism for maintaining ecosystem stability through compensatory interactions among species [59], the greater sensitivity of species diversity (mainly *α* diversity) to precipitation in desert steppe than in meadow steppe (Fig. 3B) suggests that more efforts are urgently needed to understand the effects of climate change on desert steppe for developing adaptive ecosystem management strategies. Our study has focused on species diversity composition changes along climatic gradients by excluding the grassland sites under heavy animal grazing. Human activities, mainly through animal grazing, have profound impacts on grassland species diversity [60], [61], [62]. The effects of animal grazing on species diversity along precipitation gradients in the Inner Mongolia grassland also need future studies.
